# Nicotinamide Mononucleotide (NMN) Prevents Age-Associated Transcriptional Drift in a Tissue-Dependent Manner: Mechanistic Leads From Ras-Related Protein Rab-11A-Mediated Trafficking and Carnitine Palmitoyltransferase 2-Linked Fatty Acid Oxidation

**DOI:** 10.7759/cureus.114956

**Published:** 2026-08-21

**Authors:** Ngo Cheung

**Affiliations:** 1 Psychiatry, Cheung Ngo Medical Limited, Hong Kong, HKG

**Keywords:** age-by-treatment interaction, aging, cpt2, liver, nad+ metabolism, nmn, rab11a, skeletal muscle, transcriptional drift, white adipose tissue

## Abstract

This study is a secondary computational reanalysis of the publicly available GSE85718 microarray dataset generated during the long-term nicotinamide mononucleotide (NMN) study. NMN has been reported to improve several age-sensitive physiological traits in mice, including energy metabolism, insulin sensitivity, plasma lipid profiles, and skeletal-muscle mitochondrial function. However, the transcriptional mechanisms behind these effects remain less clear than the broader nicotinamide adenine dinucleotide (NAD+) and sirtuin-centered model often used to explain NMN biology. The present analysis used a per-tissue age-by-treatment interaction model to test whether NMN modifies the rate of age-associated transcriptional change rather than simply shifting expression at one age. The model was expression ~ age × treatment, with the age-by-treatment term used as the central test. A gene was considered an NMN-rescue candidate only when it changed with age in control mice and showed an opposite-signed interaction term, consistent with NMN shifting old-age expression toward the young-control state. No individual gene reached genome-wide false discovery rate (FDR) <0.05 for age, NMN at six months, or the interaction term in skeletal muscle, liver, or white adipose tissue (WAT). Therefore, all gene-level results should be treated as hypothesis-generating. Using relaxed nominal criteria, 421 rescue candidates were identified in skeletal muscle, 355 in liver, and 397 in WAT. Only 35 genes were rescued in at least two tissues, 26 of which were direction-consistent, and none were rescued in all three tissues. Ras-related protein Rab-11A (RAB11A) emerged as the strongest cross-tissue candidate, with rescue in skeletal muscle and WAT, high confidence in at least one tissue, consistent directionality, and involvement in 39 gene set enrichment analysis (GSEA) leading-edge terms, largely related to trafficking and cellular transport. Carnitine palmitoyltransferase 2 (CPT2) was the only mitochondrial gene among the robust cross-tissue candidates and was consistently rescued in skeletal muscle and WAT, supporting a focused fatty-acid oxidation and substrate-handling hypothesis rather than broad mitochondrial activation. At the pathway level, liver showed the clearest signal: NMN was associated with suppression of fatty-acyl-coenzyme A (CoA) and long-chain fatty-acyl-CoA metabolic programs. These findings do not establish that NMN prevents transcriptional aging or that RAB11A or CPT2 mediates the physiological effects of NMN. Instead, they identify tissue-specific, testable candidates from a secondary reanalysis of an existing animal dataset. In particular, they support moving beyond a generic "NAD+ improves mitochondria" model toward testable mechanisms involving cellular logistics, membrane recycling, and substrate utilization.

## Introduction

A decline in nicotinamide adenine dinucleotide (NAD+) metabolism is widely discussed as one of the biochemical features of aging. NAD+ is required for redox reactions, but it also serves as a substrate for enzymes involved in stress responses, deoxyribonucleic acid (DNA) repair, mitochondrial homeostasis, inflammation, and chromatin regulation. This has made NAD+ biology a major focus in aging research. Nicotinamide mononucleotide (NMN) and nicotinamide riboside (NR) are often described as NAD+ precursors with the potential to improve healthspan-related phenotypes in preclinical models. The present study does not provide new animal or wet-laboratory data; it is a secondary reanalysis of an existing public microarray dataset. Reviews by Imai et al., Verdin, and Yaku et al. frame NAD+ decline as a broad aging mechanism, but they also make clear that NAD+ biology is distributed across many pathways rather than confined to a single molecular axis [1-5].

The study by Mills et al. [6] remains one of the central preclinical datasets for long-term NMN administration during normal aging. In that study, wild-type C57BL/6N mice received oral NMN at 300 mg/kg/day during aging. The authors reported suppression of age-associated body weight gain, improved energy metabolism, enhanced insulin sensitivity, improved plasma lipid profiles, better eye function, and attenuation of age-associated gene expression changes in metabolic tissues, with particular emphasis on skeletal-muscle mitochondrial oxidative metabolism. The public microarray dataset GSE85718 captures this design across skeletal muscle, liver, and white adipose tissue, with age, treatment, and tissue all represented.

That distinction matters. A simple comparison between old control mice and old NMN-treated mice can identify genes that differ at old age, but it does not establish that NMN altered the age-related trajectory. A young-age NMN effect can also be misleading, because it may reflect treatment biology that has little to do with aging. The appropriate statistical question is whether the age-associated slope differs by treatment. Thus, the design tests whether the rate of age-related transcript change differs under NMN rather than merely testing expression differences at a single time point.

If a gene rises with age in control mice and the interaction term is negative, or if a gene falls with age in control mice and the interaction term is positive, the result is consistent with NMN shifting old-age expression toward the young-control state. In this article, an "NMN-rescue candidate" refers to a gene meeting this sign-reversal pattern together with nominal evidence for both the control aging effect and the age-by-treatment interaction. For example, a gene that falls with age in control mice but has a positive interaction coefficient would be considered a candidate for NMN-associated attenuation of that age-related decline. The broader concept of age-associated transcriptional drift has also been described in other biological systems, although its magnitude and direction can vary by tissue and cell type [7].

This approach also addresses several problems that limit translation in the NAD+ precursor field. First, mechanistic models are often too simple. A common narrative is that NAD+ increases sirtuin activity, activates peroxisome proliferator-activated receptor gamma coactivator 1-alpha (PGC-1α)-like programs, and thereby improves mitochondria. That model is useful, but it is unlikely to explain all tissue-specific effects of NMN. Second, tissue heterogeneity remains poorly resolved. Skeletal muscle, liver, and adipose tissue differ sharply in function, metabolic load, cell composition, and transcriptional response to aging. A single universal transcriptional rescue program would therefore be biologically surprising. Third, the field lacks reliable molecular biomarkers of NMN response. If NAD+ precursors are to move toward precision use, pharmacodynamic markers will be needed to show that the intervention engages relevant biology in a given tissue or patient group. Finally, it remains difficult to link transcriptional changes to functional outcomes such as insulin sensitivity, lipid handling, mitochondrial oxidation, and cellular homeostasis.

The present analysis was designed with those limitations in mind. It uses GSE85718 to test whether NMN modifies age-associated gene expression changes in metabolic tissues. It then asks whether any rescue candidates are robust across tissues and whether those candidates point to plausible mechanisms. The working hypothesis was that NMN would not reverse a single shared transcriptional aging program in all tissues. Instead, it was expected to produce tissue-dependent rescue signatures that converge on metabolic function. Based on the downstream results, the strongest mechanistic leads were RAB11A-linked vesicular trafficking and CPT2-linked fatty acid oxidation.

## Materials and methods

Dataset and experimental design

**Figure 1:**
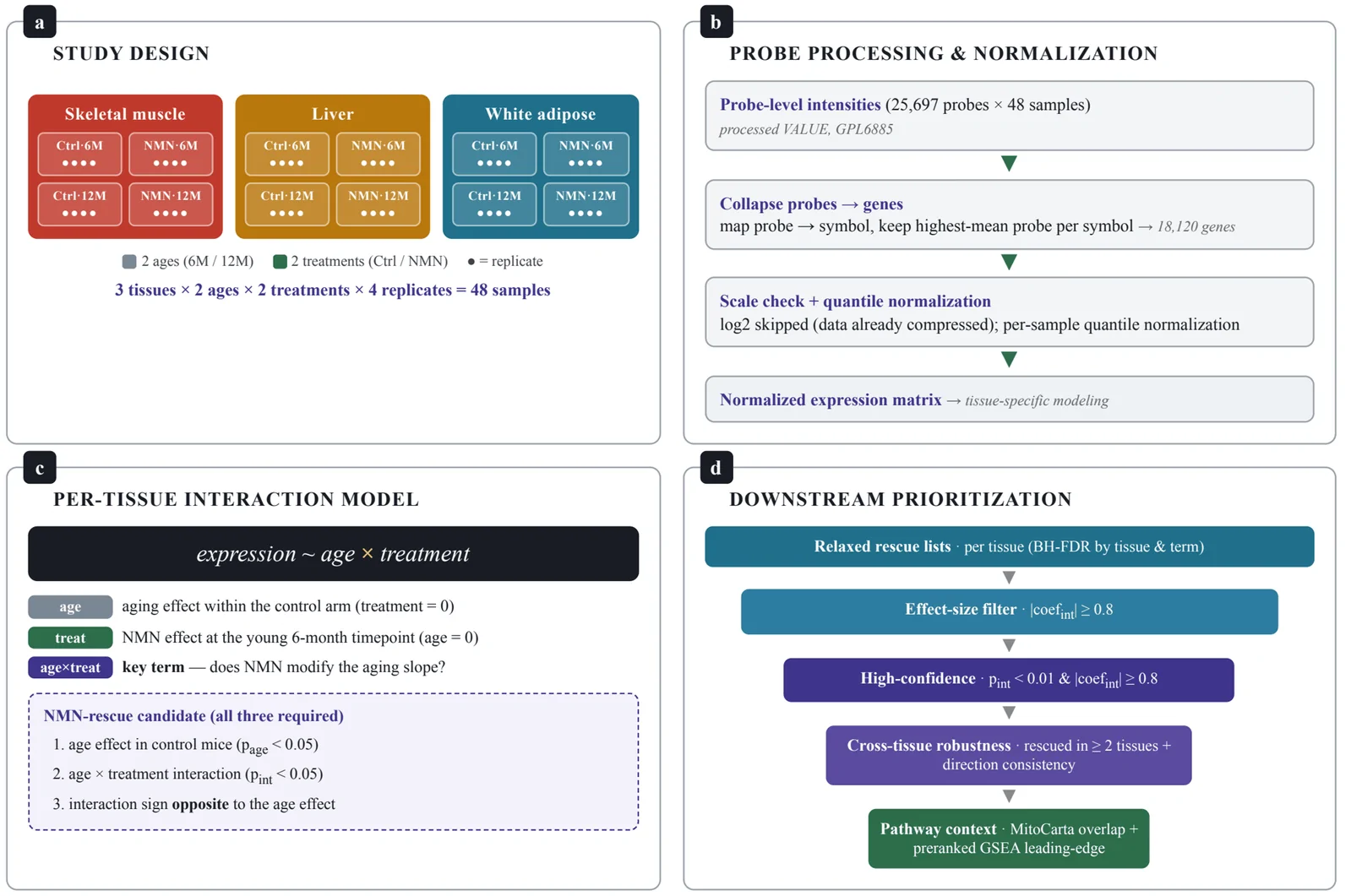
Schematic of the analysis pipeline (a) The GSE85718 design comprises 48 microarray samples spanning three metabolic tissues, two ages, two treatments, and four biological replicates per cell. (b) Probe-level data are collapsed to gene level by retaining the highest-mean probe per symbol and then quantile-normalized to yield the matrix used for modeling. (c) Within each tissue, expression is fit to an age × treatment linear model; the age-by-treatment interaction is the central test, and rescue candidates require an age effect plus an opposite-signed interaction. (d) Candidate lists are sequentially filtered by interaction effect size, statistical confidence, cross-tissue robustness with direction consistency, and pathway context via MitoCarta overlap and preranked gene set enrichment analysis (GSEA) leading-edge analysis. The schematic depicts the computational workflow only; no results are shown, and all proposed mechanistic routes remain hypotheses requiring independent validation. Image created by the author using PowerPoint (Microsoft® Corp., Redmond, WA).

The dataset analyzed here was GSE85718, generated from the long-term NMN study by Mills et al. [6] and deposited in the Gene Expression Omnibus (GEO) [8]. The analysis was a secondary reanalysis of processed public data and did not involve new animal experiments, treatment assignments, sample collection, or wet-laboratory measurements. The dataset included 48 samples across three tissues: skeletal muscle, liver, and white adipose tissue (WAT) (Figure 1). Within each tissue, samples represented two ages, 6 months and 12 months; two treatments, control and NMN; and four biological replicates per age-by-treatment cell. The microarray platform was GPL6885, the Illumina MouseRef-8 v2.0 expression beadchip.

Expression processing and normalization

Probe-level expression data were downloaded from GEO using GEOparse. Probes were mapped to gene symbols using the GPL6885 platform annotation and collapsed to gene-level values by retaining the highest-mean probe for each symbol. This reduced 25,697 probes to 18,120 genes.

The analysis used the processed GEO VALUE field rather than raw intensity files. The values appeared to be on a compressed or log-like scale because the maximum observed value did not exceed the threshold used in the analysis script to identify linear-scale intensities; accordingly, no additional log2 transformation was applied. Per-sample quantile normalization was then performed. This preprocessing decision was based on the available processed matrix and was not supported by a separate array-density, MA-plot, or raw-intensity diagnostic analysis. The resulting normalized matrix was used for all tissue-specific modeling.

No arrays were excluded as outliers, and no array weighting, surrogate-variable adjustment, batch correction, or cell-composition adjustment was performed. These omissions should be considered when interpreting the small-sample interaction results.

Statistical model

For each tissue, gene expression was modeled as expression ~ age × treatment. Age was encoded as 0 for 6 months and 1 for 12 months. Treatment was encoded as 0 for control and 1 for NMN. The age coefficient therefore estimated the age effect within the control group. The treatment coefficient estimated the NMN effect at six months. The interaction coefficient estimated whether NMN changed the aging slope. This interaction term was the main coefficient of interest because it directly tests whether NMN modifies age-related transcriptional change.

Ordinary least squares (OLS) was used because the primary scientific question was the age-by-treatment interaction coefficient itself and because the small per-cell sample size (n = 4) already limits power. The primary analysis therefore did not apply empirical-Bayes variance moderation. This choice provides a straightforward coefficient-level interpretation but may result in less stable variance estimates than moderated microarray models such as limma. A limma-based sensitivity analysis was not performed in the present computational run; therefore, equivalence between the OLS results and empirical-Bayes-moderated results cannot be claimed. The absence of this sensitivity analysis is an additional limitation and supports treating the candidate lists as hypothesis-generating.

Definition of NMN-rescue candidates

An NMN-rescue candidate was defined by three criteria. First, the gene had to show an age effect in control mice. Second, the gene had to show an age-by-treatment interaction. Third, the interaction coefficient had to have the opposite sign from the control aging coefficient. This sign-reversal criterion distinguishes a general NMN effect from a pattern consistent with NMN-associated attenuation of age-related expression drift. Because no genes passed strict genome-wide false discovery rate (FDR) thresholds, the rescue lists were generated using relaxed nominal criteria: P_age, the P value for the age effect in control mice, <0.05; P_interaction, the P value for the age-by-treatment interaction, <0.05; and opposite sign between the age effect and interaction term. These uncorrected nominal thresholds create a substantial false-positive risk, particularly when many thousands of genes are tested. The relaxed lists were used for candidate generation only and not for confirmatory inference.

Multiple testing was addressed using the Benjamini-Hochberg FDR procedure, computed by tissue and statistical term [9]. The use of a linear model follows the broad framework of microarray differential-expression modeling, although the present pipeline used OLS rather than empirical-Bayes moderation [10,11].

Downstream prioritization

Downstream prioritization proceeded in several steps. First, the relaxed rescue lists were filtered by interaction effect size using an absolute interaction coefficient threshold of 0.8. Second, high-confidence candidates were defined as genes with P_interaction <0.01 and an absolute interaction coefficient of at least 0.8. These high-confidence labels indicate stronger candidates within the nominal-P framework; they do not indicate genome-wide statistical significance. Third, cross-tissue robustness was assessed by identifying genes rescued in at least two tissues. Fourth, direction consistency was evaluated by asking whether the interaction coefficients shared the same sign across tissues (Appendix 1).

Fifth, mitochondrial overlap was assessed using a MitoCarta-informed approach. Because the full mouse MitoCarta3.0 list was not available in the run environment, a curated fallback mitochondrial set of 66 symbols was used. The exact fallback list is embedded in the analysis script used for this study. Because this list is incomplete relative to the full MitoCarta3.0 mouse annotation, all mitochondrial overlap and enrichment findings are provisional. MitoCarta3.0 remains the preferred reference because it provides an updated mitochondrial proteome with sub-organelle localization and pathway annotations [12].

Gene set enrichment analysis (GSEA)

GSEA was performed using preranked interaction coefficients [13]. Genes were ranked by the raw, unscaled NMN-by-age interaction coefficient. No coefficient standardization or additional scaling was applied. Preranked GSEA used 1,000 permutations, a minimum gene-set size of 10, and a maximum gene-set size of 500. The ranking asked which pathways were associated with positive or negative NMN-by-age interaction effects.

Gene Ontology (GO) Biological Process 2021 and the Kyoto Encyclopedia of Genes and Genomes (KEGG) 2019 Mouse were treated as the more reliable mouse-oriented libraries. Hallmark gene sets were interpreted more cautiously because they are human-derived and required upper-casing mouse gene symbols as an approximate orthology proxy. Leading-edge overlap was then used to identify robust genes that appeared repeatedly within pathway-driving subsets. Leading-edge membership was used as a prioritization heuristic and not as independent evidence that the corresponding pathway or cellular process changed functionally.

Software and code availability

The pipeline was implemented in Python and used GEOparse for GEO retrieval, pandas and NumPy for data handling, SciPy for statistical calculations, statsmodels for Benjamini-Hochberg correction, scikit-learn for quantile normalization, matplotlib and seaborn for visualization, and GSEApy for preranked GSEA (Appendix 2). The primary model was implemented with vectorized ordinary least-squares calculations, and false-discovery correction used the statsmodels multiple-testing functions. Exact package-version records were not captured during the original computational run. The complete annotated analysis script and generated processing outputs should therefore be retained with the study files to support exact rerunning of the analysis.

## Results

Overview of statistical power and gene-level significance

The first and most important result is negative: no individual genes reached genome-wide FDR <0.05 for the age effect in control mice, the NMN effect at six months, or the age-by-treatment interaction term in any of the three tissues. This was true for skeletal muscle, liver, and WAT. Accordingly, the analysis does not provide definitive gene-level discoveries, and all subsequent candidate lists are hypothesis-generating under relaxed nominal criteria.

The relaxed rescue criteria produced sizable tissue-specific lists. Skeletal muscle had 421 nominal NMN-rescue candidates. Liver had 355. White adipose tissue had 397. These numbers should not be read as the number of statistically confirmed NMN-rescued genes. They are the number of genes that met the relaxed candidate definition: nominal age effect in control mice, nominal interaction effect, and opposite sign between aging and interaction coefficients. The distinction is central to the interpretation of the entire analysis.

The downstream stringency filters reduced these lists substantially. After applying the interaction effect-size threshold of 0.8, 180 genes remained in skeletal muscle, 171 in liver, and 135 in white adipose tissue. When the high-confidence rule was applied, using P_interaction <0.01 and an absolute interaction coefficient of at least 0.8, the counts dropped to 54 genes in skeletal muscle, 49 in liver, and 36 in white adipose tissue. These high-confidence genes remain nominally selected candidates rather than genome-wide FDR-significant findings, as summarized in Table 1.

**Table 1:** Differential expression overview and rescue gene tiers Note: Effect-filtered genes were selected using an absolute interaction coefficient ≥0.8. High-confidence genes additionally required P_interaction <0.01. High-confidence status does not imply genome-wide FDR significance. NMN, nicotinamide mononucleotide; FDR, false discovery rate; P_age, P value for the age effect in control mice; P_interaction, P value for the age-by-treatment interaction

Tissue	Age effect, FDR <0.05	NMN at 6 months, FDR <0.05	Interaction, FDR <0.05	Relaxed rescue genes	Effect-filtered rescue genes	High-confidence genes	Selection mode
Skeletal muscle	0	0	0	421	180	54	Relaxed nominal P_age <0.05, P_interaction <0.05, opposite sign
Liver	0	0	0	355	171	49	Relaxed nominal P_age <0.05, P_interaction <0.05, opposite sign
WAT	0	0	0	397	135	36	Relaxed nominal P_age <0.05, P_interaction <0.05, opposite sign

Tissue-specific rescue patterns

Skeletal muscle showed the largest relaxed rescue list, with 421 genes. Of these, 178 rose with age in control mice and were lowered by NMN, whereas 243 fell with age and were raised by NMN. The skeletal-muscle pathway results were broad and noisy. Positive interaction-ranked GSEA terms included regulation of protein transport, multivesicular body organization, cytoplasmic transport-related themes, and some apoptotic and microRNA-associated terms. However, the FDR values for the top skeletal muscle terms were high, so these signals should not be treated as statistically strong pathway discoveries. They are better viewed as directional clues.

Liver showed 355 relaxed rescue candidates. Of these, 164 rose with age and were lowered by NMN, while 191 fell with age and were raised by NMN. Liver produced the clearest pathway-level signal in the entire reanalysis. NMN was associated with negative enrichment of fatty-acyl-coenzyme A (CoA) biosynthetic and metabolic processes. The strongest term was fatty-acyl-CoA biosynthetic process, with a normalized enrichment score (NES) of -1.973 and FDR 0.0109. Long-chain fatty-acyl-CoA metabolic process had NES -1.879 and FDR 0.0343, and fatty-acyl-CoA metabolic process had NES -1.858 and FDR 0.0423. KEGG fatty acid elongation also showed negative enrichment, with NES -1.774 and FDR 0.0567. This pattern suggests suppression of an age-associated hepatic lipid remodeling program. It is the most statistically credible pathway signal in the analysis.

White adipose tissue had 397 relaxed rescue candidates. Of these, 193 rose with age in control mice and were lowered by NMN, and 204 fell with age and were raised by NMN. The WAT pathway results pointed toward trafficking, ubiquitination, and metabolic signaling, but most were exploratory. The strongest negative terms included retrograde transport from endosome to Golgi, with NES -1.827 and FDR 0.0722, and protein K63-linked ubiquitination, with NES -1.808 and FDR 0.0593. Regulation of receptor-mediated endocytosis also appeared among the negative terms but with weaker FDR support. Positive terms included regulation of Ras protein signal transduction, regulation of target of rapamycin complex 1 (TORC1) signaling, and regulation of generation of precursor metabolites and energy, again with FDR values that call for caution. The directions of these rescue patterns are summarized in Table 2, and the pathway-level GSEA highlights are summarized in Table 3.

**Table 2:** Rescue gene direction within each tissue Note: These categories were generated using relaxed nominal criteria and should not be interpreted as confirmed transcriptional rescue events. WAT, white adipose tissue; NMN, nicotinamide mononucleotide

Tissue	Total relaxed rescue genes	Rose with age in controls; NMN lowered expression	Fell with age in controls; NMN raised expression
Skeletal muscle	421	178	243
Liver	355	164	191
WAT	397	193	204

**Table 3:** GSEA highlights from the interaction ranking Note: Positive NES indicates pathways whose old-age expression was raised by NMN relative to control. Negative NES indicates pathways whose old-age expression was lowered by NMN relative to control. Leading-edge representation and pathway direction were used for prioritization and do not establish functional pathway activation or suppression. GSEA, gene set enrichment analysis; NES, normalized enrichment score; FDR, false discovery rate; KEGG, Kyoto Encyclopedia of Genes and Genomes; ER, endoplasmic reticulum; miRNA, microRNA; TORC1, target of rapamycin complex 1; RNA, ribonucleic acid; WAT, white adipose tissue

Tissue	Direction	Term	NES	P	FDR
Skeletal muscle	Positive	Extrinsic apoptotic signaling pathway via death domain receptors	+1.754	0.000	0.841
Skeletal muscle	Positive	Negative regulation of developmental growth	+1.721	0.000	0.792
Skeletal muscle	Positive	Regulation of protein transport	+1.719	0.000	0.550
Skeletal muscle	Positive	Negative regulation of neuron projection development	+1.718	0.000	0.423
Skeletal muscle	Positive	Production of miRNAs involved in gene silencing by miRNA	+1.706	0.004	0.420
Skeletal muscle	Positive	Negative regulation of phosphorylation	+1.692	0.000	0.453
Skeletal muscle	Positive	Cellular response to ionizing radiation	+1.686	0.000	0.432
Skeletal muscle	Positive	Phosphatidylethanolamine metabolic process	+1.679	0.002	0.431
Skeletal muscle	Positive	Multivesicular body organization	+1.676	0.002	0.401
Skeletal muscle	Positive	Regulation of vascular endothelial growth factor receptor signaling pathway	+1.672	0.000	0.389
Skeletal muscle	Negative	Diol biosynthetic process	-1.806	0.000	0.370
Skeletal muscle	Negative	Glycogen metabolic process	-1.775	0.000	0.366
Skeletal muscle	Negative	Oxoacid metabolic process	-1.764	0.000	0.303
Skeletal muscle	Negative	Aminoglycan biosynthetic process	-1.749	0.000	0.302
Skeletal muscle	Negative	Regulation of proteasomal protein catabolic process	-1.742	0.000	0.279
Skeletal muscle	Negative	Purine nucleotide metabolic process	-1.737	0.002	0.253
Skeletal muscle	Negative	Sphingosine metabolic process	-1.735	0.000	0.227
Skeletal muscle	Negative	Glycosaminoglycan biosynthetic process	-1.708	0.000	0.311
Skeletal muscle	Negative	Regulation of telomerase RNA localization to Cajal body	-1.700	0.000	0.315
Skeletal muscle	Negative	Positive regulation of telomerase RNA localization to Cajal body	-1.665	0.004	0.490
Liver	Positive	Endoplasmic reticulum organization	+1.859	0.000	0.156
Liver	Positive	Protein insertion into ER membrane	+1.836	0.000	0.116
Liver	Positive	Spliceosomal complex assembly	+1.819	0.000	0.114
Liver	Positive	Peptidyl-serine dephosphorylation	+1.814	0.000	0.097
Liver	Positive	Regulation of lipid catabolic process	+1.797	0.000	0.111
Liver	Positive	Cortical cytoskeleton organization	+1.790	0.000	0.105
Liver	Positive	Inner mitochondrial membrane organization	+1.779	0.002	0.113
Liver	Positive	Positive regulation of sphingolipid biosynthetic process	+1.753	0.000	0.145
Liver	Positive	Positive regulation of ceramide biosynthetic process	+1.753	0.000	0.145
Liver	Positive	Regulation of ceramide biosynthetic process	+1.749	0.000	0.140
Liver	Negative	Fatty-acyl-CoA biosynthetic process	-1.973	0.000	0.011
Liver	Negative	Global genome nucleotide-excision repair	-1.889	0.000	0.041
Liver	Negative	Long-chain fatty-acyl-CoA metabolic process	-1.879	0.000	0.034
Liver	Negative	Fatty-acyl-CoA metabolic process	-1.858	0.000	0.042
Liver	Negative	Acyl-CoA metabolic process	-1.788	0.002	0.143
Liver	Negative	KEGG fatty acid elongation	-1.774	0.002	0.057
Liver	Negative	Long-chain fatty-acyl-CoA biosynthetic process	-1.753	0.000	0.238
Liver	Negative	Positive regulation of transcription from RNA polymerase II promoter involved in cellular response to chemical stimulus	-1.736	0.000	0.274
Liver	Negative	Negative regulation of response to DNA damage stimulus	-1.730	0.002	0.269
Liver	Negative	Positive regulation of protein dephosphorylation	-1.714	0.000	0.320
WAT	Positive	Negative regulation of cell cycle G2/M phase transition	+1.765	0.000	0.186
WAT	Positive	Positive regulation of Ras protein signal transduction	+1.754	0.002	0.123
WAT	Positive	Negative regulation of G2/M transition of mitotic cell cycle	+1.753	0.000	0.083
WAT	Positive	Regulation of cellular amine metabolic process	+1.710	0.002	0.180
WAT	Positive	Regulation of TORC1 signaling	+1.699	0.000	0.184
WAT	Positive	KEGG choline metabolism in cancer	+1.693	0.000	0.086
WAT	Positive	Regulation of generation of precursor metabolites and energy	+1.681	0.000	0.233
WAT	Positive	Transcription by RNA polymerase III	+1.678	0.000	0.211
WAT	Positive	Amino acid import across plasma membrane	+1.662	0.002	0.259
WAT	Positive	Negative regulation of ubiquitin-dependent protein catabolic process	+1.648	0.004	0.303
WAT	Negative	Retrograde transport, endosome to Golgi	-1.827	0.000	0.072
WAT	Negative	Protein K63-linked ubiquitination	-1.808	0.000	0.059
WAT	Negative	Regulation of receptor-mediated endocytosis	-1.730	0.002	0.239
WAT	Negative	Regulation of cellular response to transforming growth factor beta stimulus	-1.680	0.002	0.498
WAT	Negative	Vacuolar acidification	-1.660	0.008	0.568
WAT	Negative	Response to organophosphorus	-1.660	0.000	0.477
WAT	Negative	Regulation of transmembrane receptor protein serine/threonine kinase signaling pathway	-1.649	0.004	0.494
WAT	Negative	Modulation by host of symbiont process	-1.604	0.009	0.944
WAT	Negative	Histone H3-K4 methylation	-1.603	0.004	0.855
WAT	Negative	Tetrapyrrole metabolic process	-1.601	0.016	0.790

Cross-tissue robust genes

The rescue programs were mostly tissue-specific. Pairwise overlap was modest: skeletal muscle and WAT shared 15 genes, liver and skeletal muscle shared 10, and liver and WAT shared 10. No gene was rescued in all three tissues. This finding argues against a universal NMN transcriptional rescue program across these metabolic organs, as summarized in Table 4.

**Table 4:** Pairwise overlap of relaxed NMN-rescue genes Note: No gene was rescued in all three tissues. Because these sets were generated using nominal P values, the overlap counts should be interpreted as exploratory rather than as estimates of confirmed biological concordance. WAT, white adipose tissue; NMN, nicotinamide mononucleotide

Tissue set	Skeletal muscle	Liver	WAT
Skeletal muscle	421	10	15
Liver	10	355	10
WAT	15	10	397

A total of 35 genes were rescued in at least two tissues. The two principal named candidates were Ras-related protein Rab-11A (RAB11A) and carnitine palmitoyltransferase 2 (CPT2); the remaining entries are retained as official mouse gene symbols. The robust set consisted of 0610009O03RIK, 0610012A05RIK, 1700024D23RIK, 2810405F18RIK, 2810441K11RIK, 6330581L23RIK, ACD, APBA2, BCHE, CMA2, CPT2, D430042O09RIK, DUSP11, EIF3S4, FMO2, GALR1, HS3ST6, LOC192950, MAP2K2, MOBKL2C, MYO15, MYOM2, OLFR508, OLFR627, PKNOX1, PSME3, RAB11A, RANGAP1, SLC13A5, SPG21, SYNGR2, TRP53INP2, V1RE1, WAP, and ZFAND2B. The cross-tissue robust genes are listed in Table 5.

**Table 5:** Cross-tissue robust genes rescued in at least two tissues Note: Gene entries are shown using official mouse gene symbols. Priority score = 2 × number of tissues + 2 × high-confidence status + 1 × mitochondrial status + 1 × GSEA lead-driver status + 1 × direction consistency. The priority score is an exploratory ranking tool and is not a statistical measure of evidence. High-magnitude Olfr, RIK, and LOC entries should be considered lower-priority candidates for experimental follow-up because of potential probe cross-hybridization and annotation uncertainty. WAT, white adipose tissue; NA, not applicable because the gene was not rescued in that tissue; Coef., interaction coefficient; GSEA, gene set enrichment analysis; P, P value

Gene	Tissues	Coef. skeletal muscle	Coef. liver	Coef. WAT	Mean interaction	Direction consistent	High confidence	Mito- chondrial	GSEA lead terms	Best p	Priority
RAB11A	Skeletal muscle; WAT	+4.017	NA	+0.464	+2.241	Yes	Yes	No	39	2.01e-03	8
GALR1	Liver; WAT	NA	+5.007	-4.884	+0.062	No	Yes	No	2	5.00e-12	7
CMA2	Liver; WAT	NA	+5.386	+6.181	+5.783	Yes	Yes	No	0	5.99e-03	7
MYO15	Skeletal muscle; WAT	+5.990	NA	+6.091	+6.040	Yes	Yes	No	0	6.74e-03	7
OLFR627	Skeletal muscle; WAT	+3.915	NA	+3.841	+3.878	Yes	Yes	No	0	8.52e-03	7
D430042O09RIK	Liver; WAT	NA	+3.945	+3.814	+3.879	Yes	Yes	No	0	9.47e-03	7
CPT2	Skeletal muscle; WAT	+0.259	NA	+0.142	+0.201	Yes	No	Yes	4	5.32e-03	7
OLFR508	Skeletal muscle; WAT	+4.151	NA	-5.906	-0.878	No	Yes	No	0	6.39e-03	6
V1RE1	Skeletal muscle; Liver	+6.116	-3.794	NA	+1.161	No	Yes	No	0	7.78e-03	6
MAP2K2	Liver; WAT	NA	-0.072	-0.104	-0.088	Yes	No	No	4	4.61e-03	6
ACD	Liver; WAT	NA	-0.081	-0.082	-0.082	Yes	No	No	1	6.91e-03	6
WAP	Skeletal muscle; Liver	-3.722	-5.066	NA	-4.394	Yes	No	No	1	1.11e-02	6
ZFAND2B	Liver; WAT	NA	-0.065	-0.065	-0.065	Yes	No	No	2	1.23e-02	6
SLC13A5	Liver; WAT	NA	+5.190	+4.909	+5.049	Yes	No	No	5	1.62e-02	6
APBA2	Skeletal muscle; Liver	+3.449	+5.047	NA	+4.248	Yes	No	No	3	1.78e-02	6
SYNGR2	Skeletal muscle; WAT	-0.209	NA	-0.151	-0.180	Yes	No	No	1	2.72e-02	6
RANGAP1	Liver; WAT	NA	+0.137	-0.131	+0.003	No	No	No	5	4.96e-03	5
HS3ST6	Skeletal muscle; WAT	-3.894	NA	+4.690	+0.398	No	No	No	3	1.11e-02	5
MYOM2	Skeletal muscle; WAT	+3.636	NA	-3.659	-0.011	No	No	No	5	1.12e-02	5
1700024D23RIK	Skeletal muscle; Liver	+3.706	+3.937	NA	+3.821	Yes	No	No	0	1.20e-02	5
0610012A05RIK	Skeletal muscle; Liver	+3.795	+5.151	NA	+4.473	Yes	No	No	0	1.25e-02	5
6330581L23RIK	Skeletal muscle; WAT	+0.323	NA	+0.311	+0.317	Yes	No	No	0	1.41e-02	5
0610009O03RIK	Skeletal muscle; WAT	-0.112	NA	-0.081	-0.096	Yes	No	No	0	1.52e-02	5
SPG21	Skeletal muscle; Liver	-0.153	-0.067	NA	-0.110	Yes	No	No	0	1.60e-02	5
MOBKL2C	Liver; WAT	NA	-0.106	-0.163	-0.134	Yes	No	No	0	1.60e-02	5
DUSP11	Skeletal muscle; WAT	-0.070	NA	-0.097	-0.084	Yes	No	No	0	1.64e-02	5
2810405F18RIK	Skeletal muscle; Liver	+0.168	+0.077	NA	+0.123	Yes	No	No	0	1.99e-02	5
PKNOX1	Skeletal muscle; WAT	-0.105	NA	-0.116	-0.110	Yes	No	No	0	2.17e-02	5
PSME3	Skeletal muscle; Liver	+0.111	+0.089	NA	+0.100	Yes	No	No	0	2.21e-02	5
TRP53INP2	Skeletal muscle; Liver	+0.147	+0.143	NA	+0.145	Yes	No	No	0	3.01e-02	5
EIF3S4	Skeletal muscle; WAT	-0.060	NA	-0.080	-0.070	Yes	No	No	0	3.67e-02	5
FMO2	Skeletal muscle; WAT	-0.224	NA	+0.184	-0.020	No	No	No	1	3.68e-02	5
2810441K11RIK	Skeletal muscle; Liver	-0.133	-0.103	NA	-0.118	Yes	No	No	0	4.13e-02	5
BCHE	Skeletal muscle; WAT	-0.191	NA	+0.358	+0.084	No	No	No	0	2.90e-03	4
LOC192950	Liver; WAT	NA	+3.776	-0.169	+1.804	No	No	No	0	1.40e-02	4

Among these 35 robust genes, 26 were direction-consistent across tissues. Eight were also high-confidence by the stage 2 criteria. Only one robust gene was mitochondrial by the fallback mitochondrial annotation: CPT2. The mitochondrial overlap among robust genes was not statistically significant, with a hypergeometric P value of 0.109. At the tissue level, the fallback mitochondrial overlap identified CPT2, CS, and SUCLA2 in skeletal muscle; NDUFV1, POLG, and TFB2M in liver; and CPT2 in WAT.

The nonsignificant hypergeometric result does not support broad mitochondrial transcriptional restoration as a principal mechanism of NMN in this dataset. CPT2 is highlighted because it was the only mitochondrial gene among the robust cross-tissue candidates and because its biological function is directly relevant to fatty-acid substrate handling. It should not be interpreted as evidence of generalized mitochondrial biogenesis or broad mitochondrial gene-set activation, as summarized in Table 6.

**Table 6:** Mitochondrial overlap and enrichment Note: Gene entries are shown using official mouse gene symbols. Mitochondrial annotation used the fallback curated core of 66 symbols. The hypergeometric P value for the robust set was nonsignificant. Because the fallback set is incomplete relative to full MitoCarta3.0, the negative enrichment result remains provisional pending reanalysis with the complete mouse annotation. WAT, white adipose tissue; CPT2, carnitine palmitoyltransferase 2; CS, citrate synthase; SUCLA2, succinyl-CoA ligase beta subunit; NDUFV1, NADH dehydrogenase [ubiquinone] flavoprotein 1; POLG, DNA polymerase gamma catalytic subunit; TFB2M, transcription factor B2, mitochondrial; CoA, coenzyme A; P, P value

Level	Tissue or gene set	Rescue genes	Mitochondrial hits	Mitochondrial genes	Hypergeometric p
Tissue	Skeletal muscle	421	3	CPT2, CS, SUCLA2	0.162087
Tissue	Liver	355	3	NDUFV1, POLG, TFB2M	0.112503
Tissue	WAT	397	1	CPT2	0.734318
Robust set	Genes rescued in at least two tissues	35	1	CPT2	0.1093

RAB11A as the strongest cross-tissue candidate

RAB11A was the highest-priority robust gene in the final summary. It was rescued in skeletal muscle and WAT, with consistent positive interaction coefficients. In skeletal muscle, the control aging coefficient was -4.165, and the interaction coefficient was +4.017, with P_interaction = 0.00725. In WAT, the control aging coefficient was -0.408, and the interaction coefficient was +0.464, with P_interaction = 0.00201. Thus, in both tissues RAB11A fell with age in control mice and was shifted upward by NMN. It met high-confidence criteria in at least one tissue and appeared in 39 GSEA leading-edge terms, mostly in skeletal muscle.

The pathway context is important. RAB11A appeared in leading-edge terms related to multivesicular body organization and assembly, cytoplasmic microtubule organization, regulation of transport, protein localization to organelle, and plasma membrane-bounded cell projection assembly. These leading-edge appearances are exploratory and should be regarded as a prioritization heuristic rather than independent evidence that endocytic recycling changed functionally. The repeated appearance of RAB11A in transport-linked leading edges nevertheless makes the candidate biologically coherent.

Endocytic recycling controls the composition of the plasma membrane by returning internalized proteins and lipids back to the cell surface after sorting in endosomes. Grant et al. [14] emphasized that recycling pathways are spatially and temporally regulated and are important for processes such as cytokinesis, cell adhesion, morphogenesis, cell fusion, learning, and memory. More broadly, Rab guanosine triphosphatases coordinate membrane identity, vesicle budding, motility, and fusion [15]. In that context, RAB11A provides a plausible link between NMN response and cellular logistics.

The translational interpretation is that NMN may be associated with preservation of membrane trafficking or recycling capacity during aging in selected tissues. This could affect receptor availability, membrane protein quality control, and organelle-associated transport. It may also connect indirectly to insulin sensitivity, because cell-surface receptor trafficking is a key determinant of signaling competence. Rab11 has been associated with glucose transporter 4-containing vesicles and insulin-responsive trafficking, although that specific insulin-receptor link was not tested in this dataset [16]. RAB11A is therefore a testable candidate for follow-up, not a confirmed mediator of NMN action.

CPT2 as the main fatty-acid oxidation candidate

CPT2 was the only mitochondrial gene among the 35 robust cross-tissue rescue candidates. It was rescued in skeletal muscle and WAT, and the direction was consistent. In skeletal muscle, CPT2 had a control aging coefficient of -0.194 and an interaction coefficient of +0.259, with P_interaction = 0.0147. In WAT, it had a control aging coefficient of -0.088 and an interaction coefficient of +0.142, with P_interaction = 0.00532. The effect sizes were modest and did not meet the high-confidence effect threshold, but the consistency across two fat-oxidizing tissues makes CPT2 biologically compelling.

CPT2 is part of the carnitine-dependent system required for mitochondrial long-chain fatty-acid oxidation. Long-chain fatty acids must be converted and transported into mitochondria before β-oxidation can proceed efficiently. Longo et al. [17] described the carnitine system as essential for fatty-acid oxidation, and Zammit [18] framed carnitine palmitoyltransferase biology as central to metabolic function. The broader physiology of mitochondrial fatty-acid β-oxidation also supports interpreting CPT2 as a substrate-utilization candidate rather than as evidence of generalized mitochondrial activation [19]. The present data therefore support a narrow carnitine-shuttle and fatty-acid oxidation hypothesis, not a claim of broad mitochondrial transcriptional reprogramming.

CPT2 provides a direct bridge between the transcriptional results and the metabolic phenotypes reported in the original NMN study. The interpretation should still be measured. This analysis does not show that NMN increases CPT2 protein abundance, CPT2 enzyme activity, or fatty-acid oxidation flux. It only shows that CPT2 expression met the relaxed rescue definition in two tissues and was the only robust mitochondrial gene under the fallback annotation. That makes CPT2 a high-priority validation target, not a confirmed mechanism.

MYO15, CMA2, and other high-magnitude robust candidates

MYO15 and CMA2 also stood out because of their large, direction-consistent interaction effects. MYO15 was rescued in skeletal muscle and WAT. Its mean interaction coefficient across tissues was +6.040, with low cross-tissue variability, and it met high-confidence criteria. This pattern suggests a possible connection to cytoskeletal organization or motor-protein-related biology. Such a connection could matter in muscle and adipose tissue, where cellular structure, intracellular transport, and organelle positioning are important. However, the dataset does not establish a direct role for MYO15 in NMN response.

CMA2 was rescued in liver and WAT, with a mean interaction coefficient of +5.783 and direction consistency. It also met high-confidence criteria. CMA2 may point toward peptide processing or local signaling biology in metabolic tissues, but this interpretation is tentative. As with MYO15, the effect size is notable, but the relaxed statistical framework and microarray platform make independent validation essential.

Several other robust genes were prioritized by the downstream scoring, including GALR1, OLFR627, D430042O09RIK, OLFR508, V1RE1, MAP2K2, ACD, WAP, ZFAND2B, SLC13A5, APBA2, SYNGR2, and RANGAP1. Some of these genes are biologically interpretable, while others, especially olfactory receptor (Olfr), RIK, and LOC entries, may carry a higher risk of probe or annotation noise. High-magnitude Olfr, RIK, and LOC candidates should be considered the lowest priority for wet-laboratory follow-up until probe identity, hybridization specificity, and current gene annotation are independently verified. The presence of multiple such genes is one reason the gene-level results should not be over-read.

Direction-discordant genes

Nine robust genes showed direction discordance, meaning NMN moved expression in opposite directions across tissues. These were BCHE, FMO2, GALR1, HS3ST6, LOC192950, MYOM2, OLFR508, RANGAP1, and V1RE1. GALR1 is a useful example. It was rescued in liver and WAT, but with opposite interaction signs: positive in liver and negative in WAT. This does not necessarily make the result unimportant. It means the biology is tissue-dependent and should not be summarized as a single systemic effect.

Direction discordance is translationally relevant. If NAD+ precursor responses differ across tissues, then biomarkers from one tissue may not generalize to another. It also suggests that safety and efficacy should be assessed in a tissue-aware way. A gene that appears "rescued" in two tissues may still reflect different mechanisms in each tissue.

## Discussion

Mechanistic synthesis

The central finding from this reanalysis is that NMN does not appear to reverse a universal transcriptional aging program across skeletal muscle, liver, and WAT. More precisely, the interaction-based analysis identified tissue-dependent patterns consistent with attenuation of selected age-associated expression changes, but it did not establish that NMN prevents transcriptional aging. The effects are largely tissue-specific. That conclusion is supported by the modest pairwise overlap among rescue lists and by the absence of any gene rescued in all three tissues. This is not a failure of the analysis. It is biologically plausible. These tissues differ in their metabolic duties, cell composition, exposure to lipid flux, endocrine function, and age-related stress. A single shared transcriptional rescue signature would have been surprising.

**Figure 2:**
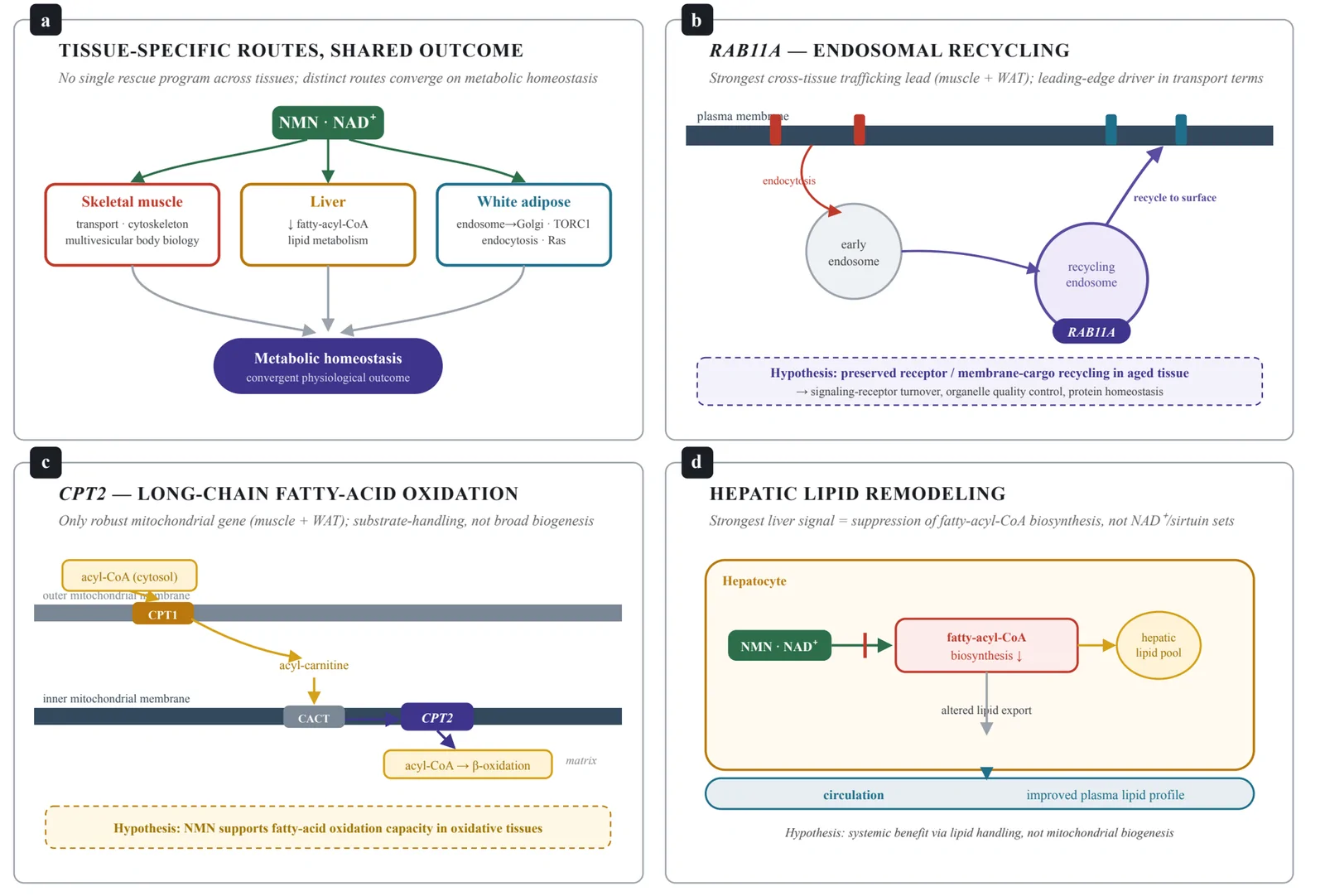
Candidate mechanistic routes through which long-term NMN may counter age-related transcriptional drift Proposed mechanistic routes by which NMN may oppose age-related transcriptional change; all panels depict hypotheses rather than measured results. (a) Rather than a single universal program, NMN engages distinct tissue-specific routes in skeletal muscle, liver, and white adipose tissue (WAT) that converge on metabolic homeostasis. (b) RAB11A, the strongest cross-tissue trafficking lead, marks recycling endosomes; its preservation could maintain receptor and membrane-cargo recycling, supporting signaling turnover and protein quality control in aged tissue. (c) CPT2, the only robust mitochondrial gene, completes the carnitine shuttle on the inner mitochondrial membrane, implicating support of long-chain fatty-acid oxidation capacity rather than broad mitochondrial biogenesis. (d) In liver, the dominant signal is suppression of fatty-acyl-CoA biosynthesis, suggesting systemic benefit through hepatic lipid remodeling and an improved plasma lipid profile. Schematics illustrate candidate mechanisms only; no quantitative findings are shown. Image created by the author using PowerPoint (Microsoft® Corp., Redmond, WA).

The stronger interpretation is that NMN may produce tissue-dependent transcriptional rescue programs that converge on related physiological outcomes (Figure 2). In skeletal muscle, the signals point toward transport, cytoskeletal organization, multivesicular body biology, and some stress-related processes, although pathway FDR support was weak. In liver, the strongest signal is suppression of fatty-acyl-CoA and long-chain fatty-acyl-CoA metabolism. In WAT, the exploratory signals involve endosome-to-Golgi transport, K63-linked ubiquitination, receptor-mediated endocytosis, Ras signaling, target of rapamycin complex 1 (TORC1) regulation, and energy precursor metabolism. These are not three copies of the same program. They look more like tissue-specific routes toward metabolic homeostasis.

RAB11A is the clearest trafficking lead. Its rescue in skeletal muscle and WAT, direction consistency, high-confidence status in at least one tissue, and repeated appearance in GSEA leading-edge terms make it the strongest cross-tissue candidate in this dataset. However, repeated GSEA leading-edge membership should be viewed as a prioritization signal rather than independent functional confirmation. The biological appeal is straightforward. Aging cells must maintain the composition of the plasma membrane while managing receptor turnover, vesicle movement, organelle communication, and protein quality control. Endocytic recycling is one of the systems that supports this maintenance. If NMN helps preserve or restore RAB11A-linked recycling, it could influence how aged metabolic tissues handle signaling proteins and membrane cargo. This remains a testable hypothesis rather than a demonstrated mechanism.

CPT2 provides a second, more metabolic lead. Its rescue in both skeletal muscle and WAT fits the original report's emphasis on improved energy metabolism and skeletal-muscle oxidative function. CPT2 does not support broad mitochondrial transcriptional activation by itself. In fact, the mitochondrial enrichment results were weak and nonsignificant for the robust set. It points instead to substrate use, especially long-chain fatty-acid oxidation, as a candidate mechanism. This distinction matters. NMN may improve metabolic function not by turning on a large mitochondrial biogenesis program across tissues, but by supporting selected mitochondrial or substrate-handling components in the tissues where they matter most.

The liver result adds another layer. The strongest pathway signal was not a broad NAD+, sirtuin, or mitochondrial gene set. It was suppression of fatty-acyl-CoA biosynthetic and metabolic processes. This may help explain the improved plasma lipid profiles reported by Mills et al. [6]. If aging is accompanied by hepatic lipid remodeling, and NMN blunts part of that transcriptional program, then the liver may contribute to systemic metabolic benefit through lipid handling rather than through a simple increase in mitochondrial gene expression.

Translational implications

The translational value of this analysis lies in prioritization. RAB11A and CPT2 are not ready to serve as clinical biomarkers. They are, however, plausible pharmacodynamic candidates for validation. RAB11A could be tested as a marker of trafficking or membrane-recycling response in metabolic tissues. CPT2 could be tested as a marker of fatty-acid oxidation capacity. Both are more mechanistically specific than measuring NAD+ alone. NAD+ abundance may show exposure or pathway engagement, but it does not necessarily show which downstream tissue functions have changed.

More broadly, NAD+ repletion has been linked to mitochondrial and stem-cell phenotypes in mice, but those findings do not establish a universal transcriptional mechanism [20]. Human evidence also remains preliminary. In a small randomized, placebo-controlled trial, NMN increased muscle insulin sensitivity in postmenopausal women with prediabetes [21]. This finding provides translational context but does not establish that RAB11A or CPT2 mediates the response.

The translational hierarchy from these results is clear. The RAB11A trafficking signature has medium-term potential as a biomarker and mechanistic target, especially if future studies connect it to receptor recycling, membrane protein turnover, or insulin signaling in aged tissue. CPT2 has medium-term potential as a metabolic readout tied to fatty-acid oxidation. The liver fatty-acyl-CoA suppression signal has short- to medium-term value because it is the strongest pathway-level result and connects naturally to circulating lipid phenotypes. The broader pattern of tissue-specific rescue supports longer-term development of precision NAD+ strategies, including tissue-targeted delivery, patient stratification, and combination approaches. Direction-discordant genes, such as GALR1, add a cautionary note: a response that looks favorable in one tissue may not have the same meaning elsewhere.

These findings also help address some of the current difficulties in the NAD+ precursor field. They challenge the overly simple idea that NAD+ boosting works mainly through a single mitochondrial biogenesis pathway. They provide evidence, although still exploratory, that trafficking and substrate utilization may be important parts of the response. They also show why tissue heterogeneity should be expected rather than treated as noise. Finally, they offer a short list of genes that can be tested directly by quantitative polymerase chain reaction (qPCR), protein assays, enzyme assays, and functional experiments.

Limitations

The limitations are substantial and should be stated plainly. First, no gene reached genome-wide FDR <0.05 for the age effect, NMN at six months, or the interaction term in any tissue. All rescue lists were therefore generated using relaxed nominal thresholds. Because the nominal p <0.05 criteria were applied across thousands of probes or genes, the false-positive risk is high, particularly for candidates selected because of large coefficients or repeated nominal appearance across tissues. This makes the analysis hypothesis-generating. It does not establish definitive NMN-rescued genes.

Second, the sample size was small. Each tissue-by-age-by-treatment cell had four replicates. Interaction tests are demanding even in larger studies, and small n reduces power while increasing sensitivity to outliers. Large interaction coefficients may reflect real biology, but they may also arise from unstable variance estimates or noise, particularly for low-signal probes. A moderated linear-model sensitivity analysis was not performed, so the effect of empirical-Bayes variance moderation on the candidate lists remains unknown.

Third, the platform was an older microarray. Probe annotation, cross-hybridization, and low-confidence gene symbols can affect interpretation. The platform may also provide incomplete coverage of lowly expressed transcripts and may produce ambiguous signals for Olfr, RIK, and LOC annotations. These high-magnitude, poorly annotated genes should therefore be considered low-priority targets for experimental validation until their probe specificity is confirmed. Gene symbols, such as RAB11A and CPT2, are more interpretable, but they are still transcript-level findings from a microarray dataset.

Fourth, the mitochondrial analysis was incomplete. The run used a fallback curated mitochondrial set of 66 symbols because the full mouse MitoCarta3.0 list was not available. This means that mitochondrial enrichment may be underestimated or distorted. The nonsignificant hypergeometric P value of 0.1093 should not be interpreted as evidence that mitochondrial involvement is absent, but it also does not support broad mitochondrial restoration. The result remains provisional pending a full MitoCarta3.0 mouse reanalysis.

Fifth, GSEA results varied in strength. Liver fatty-acyl-CoA terms had the strongest FDR support. Many skeletal-muscle and WAT terms were suggestive but not statistically strong after FDR correction. Leading-edge analysis is useful for prioritizing genes such as RAB11A, but repeated leading-edge membership is not independent evidence of endocytic recycling, trafficking, or other pathway-level functional change.

Sixth, preprocessing was based on the processed GEO expression matrix. Although the values appeared to be on a compressed or log-like scale, separate density and MA-plot diagnostics were not generated as part of the original run. The absence of these diagnostics limits independent assessment of array-scale behavior and normalization adequacy.

Finally, transcription does not equal function. The analysis cannot determine whether RAB11A protein, vesicle recycling, CPT2 activity, fatty-acid oxidation flux, or insulin receptor trafficking actually changed. Those questions require targeted experiments.

Future directions

The next step should be focused validation rather than broader gene-list interpretation. RAB11A and CPT2 should be tested by qPCR in the same tissues and, ideally, in independent NMN-treated aging cohorts. Protein-level validation should follow. For RAB11A, useful assays would include endosomal recycling, receptor recycling, membrane cargo return to the cell surface, and colocalization with recycling endosome markers. If insulin sensitivity is the functional anchor, insulin receptor or glucose transporter (GLUT)-related trafficking assays would be especially relevant. For CPT2, fatty-acid oxidation assays, acylcarnitine profiling, mitochondrial respiration with lipid substrates, and CPT2 protein or activity measurements would help determine whether the transcript-level signal has metabolic consequences.

The liver fatty-acyl-CoA program should be validated with targeted lipidomics and expression assays for enzymes involved in acyl-CoA metabolism and fatty acid elongation. Because the pathway result was stronger than most individual gene results, this may be one of the most efficient validation routes. Finally, future analyses should integrate the full MitoCarta3.0 mouse gene set, updated gene annotations, ribonucleic acid sequencing (RNA-seq) datasets when available, and human NAD+ precursor studies. A future statistical sensitivity analysis using limma with empirical-Bayes moderation would also help determine whether the current candidate priorities are stable across variance-estimation frameworks. The goal should not be to prove that NMN has one universal molecular signature. The goal should be to define which tissues respond, which pathways move, and which biomarkers track functional benefit.

## Conclusions

This interaction-based secondary reanalysis of GSE85718 identified tissue-dependent patterns consistent with NMN-associated attenuation of selected age-related transcriptional changes. It did not produce FDR-significant gene-level discoveries, and it should not be presented as definitive evidence that any single gene mediates NMN's effects. Its value is in prioritization. Under relaxed, hypothesis-generating criteria, NMN-rescue candidates were identified in skeletal muscle, liver, and WAT, but overlap across tissues was modest, and no gene was rescued in all three tissues. Within that tissue-specific landscape, RAB11A and CPT2 stand out. RAB11A is the strongest cross-tissue trafficking candidate and points toward endosomal recycling, vesicular movement, and membrane homeostasis as under-appreciated parts of the NMN response. Its repeated GSEA leading-edge representation supports prioritization but does not independently establish a change in endocytic recycling. CPT2 is the strongest fatty-acid oxidation candidate, linking the transcriptional findings to substrate utilization in skeletal muscle and WAT. The available mitochondrial analysis does not support broad mitochondrial restoration; instead, CPT2 suggests a narrower carnitine-shuttle and fatty-acid oxidation hypothesis. Liver adds the clearest pathway-level result, with suppression of fatty-acyl-CoA and fatty acid elongation programs.

The broader implication is that NAD+ precursor biology should move beyond generic claims about mitochondrial improvement. NMN may act through tissue-specific programs that include cellular logistics, lipid handling, and selected metabolic enzymes. These findings provide a practical roadmap for validation rather than proof of mechanism: confirm RAB11A and CPT2 at RNA and protein levels, test trafficking and fatty-acid oxidation functionally, re-run mitochondrial enrichment with full MitoCarta3.0, and connect tissue-specific signatures to metabolic outcomes.

## Appendices

Appendix 1

**Table 7:** Cross-tissue robust genes rescued in at least two tissues Note: Priority score = 2 × number of tissues + 2 × high-confidence status + 1 × mitochondrial status + 1 × GSEA lead-driver status + 1 × direction consistency

Gene	Tissues	Coef. skeletal muscle	Coef. liver	Coef. WAT	Mean interaction	Direction consistent	High confidence	Mitochondrial	GSEA lead terms	Best p	Priority
RAB11A	Skeletal muscle; WAT	+4.017	NA	+0.464	+2.241	Yes	Yes	No	39	2.01e-03	8
GALR1	Liver; WAT	NA	+5.007	-4.884	+0.062	No	Yes	No	2	5.00e-12	7
CMA2	Liver; WAT	NA	+5.386	+6.181	+5.783	Yes	Yes	No	0	5.99e-03	7
MYO15	Skeletal muscle; WAT	+5.990	NA	+6.091	+6.040	Yes	Yes	No	0	6.74e-03	7
OLFR627	Skeletal muscle; WAT	+3.915	NA	+3.841	+3.878	Yes	Yes	No	0	8.52e-03	7
D430042O09RIK	Liver; WAT	NA	+3.945	+3.814	+3.879	Yes	Yes	No	0	9.47e-03	7
CPT2	Skeletal muscle; WAT	+0.259	NA	+0.142	+0.201	Yes	No	Yes	4	5.32e-03	7
OLFR508	Skeletal muscle; WAT	+4.151	NA	-5.906	-0.878	No	Yes	No	0	6.39e-03	6
V1RE1	Skeletal muscle; Liver	+6.116	-3.794	NA	+1.161	No	Yes	No	0	7.78e-03	6
MAP2K2	Liver; WAT	NA	-0.072	-0.104	-0.088	Yes	No	No	4	4.61e-03	6
ACD	Liver; WAT	NA	-0.081	-0.082	-0.082	Yes	No	No	1	6.91e-03	6
WAP	Skeletal muscle; Liver	-3.722	-5.066	NA	-4.394	Yes	No	No	1	1.11e-02	6
ZFAND2B	Liver; WAT	NA	-0.065	-0.065	-0.065	Yes	No	No	2	1.23e-02	6
SLC13A5	Liver; WAT	NA	+5.190	+4.909	+5.049	Yes	No	No	5	1.62e-02	6
APBA2	Skeletal muscle; Liver	+3.449	+5.047	NA	+4.248	Yes	No	No	3	1.78e-02	6
SYNGR2	Skeletal muscle; WAT	-0.209	NA	-0.151	-0.180	Yes	No	No	1	2.72e-02	6
RANGAP1	Liver; WAT	NA	+0.137	-0.131	+0.003	No	No	No	5	4.96e-03	5
HS3ST6	Skeletal muscle; WAT	-3.894	NA	+4.690	+0.398	No	No	No	3	1.11e-02	5
MYOM2	Skeletal muscle; WAT	+3.636	NA	-3.659	-0.011	No	No	No	5	1.12e-02	5
1700024D23RIK	Skeletal muscle; Liver	+3.706	+3.937	NA	+3.821	Yes	No	No	0	1.20e-02	5
0610012A05RIK	Skeletal muscle; Liver	+3.795	+5.151	NA	+4.473	Yes	No	No	0	1.25e-02	5
6330581L23RIK	Skeletal muscle; WAT	+0.323	NA	+0.311	+0.317	Yes	No	No	0	1.41e-02	5
0610009O03RIK	Skeletal muscle; WAT	-0.112	NA	-0.081	-0.096	Yes	No	No	0	1.52e-02	5
SPG21	Skeletal muscle; Liver	-0.153	-0.067	NA	-0.110	Yes	No	No	0	1.60e-02	5
MOBKL2C	Liver; WAT	NA	-0.106	-0.163	-0.134	Yes	No	No	0	1.60e-02	5
DUSP11	Skeletal muscle; WAT	-0.070	NA	-0.097	-0.084	Yes	No	No	0	1.64e-02	5
2810405F18RIK	Skeletal muscle; Liver	+0.168	+0.077	NA	+0.123	Yes	No	No	0	1.99e-02	5
PKNOX1	Skeletal muscle; WAT	-0.105	NA	-0.116	-0.110	Yes	No	No	0	2.17e-02	5
PSME3	Skeletal muscle; Liver	+0.111	+0.089	NA	+0.100	Yes	No	No	0	2.21e-02	5
TRP53INP2	Skeletal muscle; Liver	+0.147	+0.143	NA	+0.145	Yes	No	No	0	3.01e-02	5
EIF3S4	Skeletal muscle; WAT	-0.060	NA	-0.080	-0.070	Yes	No	No	0	3.67e-02	5
FMO2	Skeletal muscle; WAT	-0.224	NA	+0.184	-0.020	No	No	No	1	3.68e-02	5
2810441K11RIK	Skeletal muscle; Liver	-0.133	-0.103	NA	-0.118	Yes	No	No	0	4.13e-02	5
BCHE	Skeletal muscle; WAT	-0.191	NA	+0.358	+0.084	No	No	No	0	2.90e-03	4
LOC192950	Liver; WAT	NA	+3.776	-0.169	+1.804	No	No	No	0	1.40e-02	4

**Table 8:** Direction-discordant robust genes

Gene	Tissues	Coef. skeletal muscle	Coef. liver	Coef. WAT	Interpretive note
BCHE	Skeletal muscle; WAT	-0.191	NA	+0.358	Opposite NMN interaction sign across tissues.
FMO2	Skeletal muscle; WAT	-0.224	NA	+0.184	Opposite NMN interaction sign across tissues.
GALR1	Liver; WAT	NA	+5.007	-4.884	High-confidence but tissue-discordant.
HS3ST6	Skeletal muscle; WAT	-3.894	NA	+4.690	Opposite NMN interaction sign across tissues.
LOC192950	Liver; WAT	NA	+3.776	-0.169	Opposite NMN interaction sign across tissues.
MYOM2	Skeletal muscle; WAT	+3.636	NA	-3.659	Opposite NMN interaction sign across tissues.
OLFR508	Skeletal muscle; WAT	+4.151	NA	-5.906	High-confidence but tissue-discordant.
RANGAP1	Liver; WAT	NA	+0.137	-0.131	Opposite NMN interaction sign across tissues.
V1RE1	Skeletal muscle; Liver	+6.116	-3.794	NA	High-confidence but tissue-discordant.

**Table 9:** Leading-edge robust-gene overlap Note: Stage 3 identified 14 robust genes appearing in GSEA leading-edge terms. RAB11A was the highest-priority candidate, appearing in 39 leading-edge terms.

Tissue	Representative leading-edge term	NES	FDR	Robust gene in leading edge
Skeletal muscle	Aminoglycan biosynthetic process	-1.749	0.302	HS3ST6
Skeletal muscle	Glycosaminoglycan metabolic process	-1.708	0.311	HS3ST6
Skeletal muscle	Multivesicular body organization	+1.676	0.401	RAB11A
Skeletal muscle	Multivesicular body assembly	+1.645	0.399	RAB11A
Skeletal muscle	Cytoplasmic microtubule organization	+1.634	0.374	RAB11A
Skeletal muscle	Regulation of transport	+1.540	0.529	RAB11A
Skeletal muscle	Protein localization to organelle	+1.318	0.673	RAB11A
Liver	Neuropeptide signaling pathway	+1.314	0.753	GALR1
Liver	Anion transport	+1.294	0.747	SLC13A5
Liver	C4-dicarboxylate transport	+1.232	0.791	SLC13A5
Liver	Dicarboxylic acid transport	+1.134	0.865	SLC13A5
Liver	Melanosome transport	-1.124	0.844	RAB11A
Liver	Nervous system development	+1.024	0.943	APBA2
WAT	Anion transmembrane transport	+1.577	0.507	SLC13A5
WAT	Plasma membrane bounded cell projection organization	+1.523	0.553	RAB11A
WAT	Aminoglycan biosynthetic process	+1.408	0.665	HS3ST6
WAT	Endocrine and other factor-regulated calcium reabsorption	+1.391	0.734	RAB11A
WAT	Organic acid transport	+1.369	0.695	SLC13A5
WAT	Cilium assembly	+1.242	0.725	RAB11A

**Table 10:** Suggested validation priorities

Priority group	Candidate or pathway	Evidence from re-analysis	Suggested validation
Highest cross-tissue lead	RAB11A	Rescued in skeletal muscle and WAT; direction-consistent; high-confidence; 39 GSEA leading-edge terms; priority score 8.	qPCR, Western blot, endosomal recycling assays, membrane cargo recycling, insulin receptor or related trafficking assays.
Mitochondrial/metabolic lead	CPT2	Only robust mitochondrial gene under fallback annotation; rescued in skeletal muscle and WAT; direction-consistent; priority score 7.	qPCR, protein abundance, CPT2 activity, fatty-acid oxidation flux, acylcarnitine profiling.
Strong pathway-level signal	Hepatic fatty-acyl-CoA metabolism	Most statistically supported GSEA signal; fatty-acyl-CoA biosynthetic process FDR = 0.011; long-chain fatty-acyl-CoA metabolic process FDR = 0.034.	Targeted lipidomics, hepatic acyl-CoA profiling, enzyme expression assays, plasma lipid correlation.
High-magnitude consistent genes	MYO15, CMA2, OLFR627, D430042O09RIK	Robust across two tissues, direction-consistent, high-confidence, large interaction coefficients.	Independent transcript validation; cautious interpretation for genes with limited mechanistic annotation.
Tissue-specific caution group	GALR1, OLFR508, V1RE1, and other direction-discordant genes	Robust in frequency but not in direction; NMN interaction sign differed across tissues.	Validate tissue-by-tissue; avoid systemic interpretation without functional confirmation.

Appendix 2

The code used for the computational reanalysis is publicly available on GitHub at: https://github.com/cheungngo/088B-Nicotinamide-Mononucleotide-NMN-Prevents-Age-Associated-Transcriptional-Drift. The repository includes the analysis notebook, “088B_GSE85718_-_NMN_Aging_Microarray_Pipeline.ipynb,” which documents the data-processing, statistical-analysis, and downstream interpretation workflow. The repository is openly accessible and does not require authentication for viewing or downloading the analysis files.
